# Diphtheria

**Published:** 1878-02

**Authors:** 


### Diphtheria—Report of the State Board of Health of California.

In the last Report of the State Board of Health of California, an interesting epidemic of diphtheria is reported as having occurred during the month of April, 1876, in the Asylum for Orphans at Vallejo. During the period referred to, there were forty-three cases and ten deaths. The board state that "one of the business men of Vallejo was called to Santa Rosa, Sonoma County, to attend the funeral of some members of a family of relatives who had died from the scourge, diphtheria. On returning to Vallejo, he and some of his family were taken down with the disease, and were attended by members of a family residing near the Home for Orphans. The children of the last-mentioned family were attending the School of the Orphans' Home at the time they were taken sick. Two of the family died, and soon after some of the Home inmates were taken sick with the same disease." During the prevalence of the epidemic no sufficient precaution was taken to isolate the sick, nor was attention apparently given to the fact of the contagiousness of the disease. The board also gives some interesting facts with regard to the transmission of diphtheria from point to point. A child just recovering from diphtheria went to visit a family at Dixon, wearing the same clothes as used during its illness. One of the children of this household was soon taken sick with the disease and died, there having been previously no diphtheria in the vicinity and no communication by the child with infected localities. Free intercourse with the sick was allowed, and the funeral of the deceased child was largely attended by other children, thereby causing an outbreak in the school near by, from which a large number of cases started. Not one person became ill who had not been exposed to direct contagion. The partial dependance of the disease upon general unsanitary conditions is noted. In San Francisco, it was most prevalent in those parts of the city where the sewerage is bad, where the soil is damp, where overcrowding, filth, and bad ventilation coexist with the other conditions usually prevailing in places occupied by the class of people who are least willing or unable to live in a manner favorable to health.—*Boston Med. and Surg. Jour.*

